# Quantification of Dexterity as the Dynamical Regulation of Instabilities: Comparisons Across Gender, Age, and Disease

**DOI:** 10.3389/fneur.2014.00053

**Published:** 2014-04-15

**Authors:** Emily L. Lawrence, Isabella Fassola, Inge Werner, Caroline Leclercq, Francisco J. Valero-Cuevas

**Affiliations:** ^1^Brain Body Dynamics Laboratory, Department of Biomedical Engineering, University of Southern California, Los Angeles, CA, USA; ^2^Institut de la Main, Clinique Jouvenet, Paris, France; ^3^Institute of Sports Science, University of Innsbruck, Innsbruck, Austria; ^4^Brain Body Dynamics Laboratory, Division of Biokinesiology and Physical Therapy, University of Southern California, Los Angeles, CA, USA

**Keywords:** sensorimotor function, rehabilitation, dexterity, hand, leg, aging, sex differences, sociobiology

## Abstract

Dexterous manipulation depends on using the fingertips to stabilize unstable objects. The Strength–Dexterity paradigm consists of asking subjects to compress a slender and compliant spring prone to buckling. The maximal level of compression [requiring low fingertip forces <300 grams force (gf)] quantifies the neural control capability to dynamically regulate fingertip force vectors and motions for a dynamic manipulation task. We found that finger dexterity is significantly affected by age (*p* = 0.017) and gender (*p* = 0.021) in 147 healthy individuals (66F, 81M, 20–88 years). We then measured finger dexterity in 42 hands of patients following treatment for osteoarthritis of the base of the thumb (CMC OA, 33F, 65.8 ± 9.7 years), and 31 hands from patients being treated for Parkinson’s disease (PD, 6F, 10M, 67.68 ± 8.5 years). Importantly, we found no differences in finger compression force among patients or controls. However, we did find stronger age-related declines in performance in the patients with PD (slope −2.7 gf/year, *p* = 0.002) than in those with CMC OA (slope −1.4 gf/year, *p* = 0.015), than in controls (slope −0.86 gf/year). In addition, the temporal variability of forces during spring compression shows clearly different dynamics in the clinical populations compared to the controls (*p* < 0.001). Lastly, we compared dexterity across extremities. We found stronger age (*p* = 0.005) and gender (*p* = 0.002) effects of leg compression force in 188 healthy subjects who compressed a larger spring with the foot of an isolated leg (73F, 115M, 14–92 years). In 81 subjects who performed the tests with all four limbs separately, we found finger and leg compression force to be significantly correlated (females ρ = 0.529, *p* = 0.004; males ρ = 0.403, *p* = 0.003; 28F, 53M, 20–85 years), but surprisingly found no differences between dominant and non-dominant limbs. These results have important clinical implications, and suggest the existence – and compel the investigation – of systemic versus limb-specific mechanisms for dexterity.

## Introduction

Dynamic upper extremity function in general, and of the fingertips in particular, is essential for activities of daily living (ADLs) and quality of life (1, 2). While there are multiple measures of hand function, we have historically lacked a means to quantify the dynamical interaction of the fingertips with objects without the confounds of strength, functional adaptations, whole-arm coordination, visual acuity, etc. We have proposed the Strength–Dexterity (SD) paradigm as a versatile, repeatable, and informative paradigm to quantify finger dexterity across the lifespan in some clinical populations. We define dexterity as the sensorimotor capability to dynamically regulate fingertip force vectors and motions to stabilize an unstable object (3–13). This paradigm consists of testing the extent to which people can compress a slender spring prone to buckling. The spring naturally becomes unstable as it is compressed; thus the maximal level of compression is indicative of the maximal sensorimotor capability to control the fingertips. The springs are designed to require very low forces to reflect the nature of ADLs. Moreover, functional magnetic resonance imaging (fMRI) studies show the SD paradigm can systematically interrogate brain function for dexterous manipulation, which exhibits differential activity across cortical networks depending on the level of difficulty and behavioral goals of the task (4, 7, 8).

Given that we have previously established the reliability and utility of this approach to dexterity (3–13), the purpose of this work is to understand the effects of gender, age, and disease on this sensorimotor ability to control instabilities. The effect of age on motor function in general, and hand function in particular, is well known (2, 13–15). However, recent studies using the SD paradigm have demonstrated its ability to detect previously unknown changes in dexterity lasting into late adolescence in typical development (6, 9, 10), or starting in middle age in healthy older adults (13). One goal of this work is to expand upon those findings by including larger numbers of participants, and including those individuals diagnosed with clinical conditions. While the effect of gender on muscle strength is well known, its effects on sensorimotor function are less clear. There continues to be keen clinical interest given the greater incidence of some musculoskeletal pathologies and injuries in women, such as osteoarthritis (16) and non-contact ligament tears (17). The literature contains contradictory reports (15, 18) that feed continued debate on the issue. Our own work using the SD paradigm has hinted at gender differences in dexterity in typical development (6, 10), but these remain to be explored in detail.

Lastly, our more recent work has extended the concept of finger dexterity to limbs in general. By simply scaling up the physical size of our test system, we have introduced the concept of limb dexterity (19). The Lower Extremity Dexterity (LED) test has been shown to be a valid and repeatable metric of dynamic leg function (19). Importantly, our report of strong differences in leg dexterity between men and women has begun to provide a neuromuscular explanation for gender differences in agility, and the much higher incidence of non-contact ligament tears in female athletes (19, 20). We are therefore compelled to explore the nature of systemic versus limb-specific dexterity as it relates to age and gender. This is necessary to further our understanding of the neural mechanisms for dynamical function in health and disease.

## Materials and Methods

All participants gave their informed consent to the experimental protocol, which was approved by the Health Sciences Campus Institutional Review Board at the University of Southern California in Los Angeles, and/or the relevant ethics committees at the Institut de la Main-Clinique Jouvenet in Paris, and the Institute of Sports Science in Innsbruck.

### Control subjects

We measured finger dexterity in 147 healthy volunteers (66F, 81M, 52.7 ± 21.6 years) between 20 and 88 years of age to use as baseline data for comparison. Similarly, we measured single leg dexterity in 188 healthy volunteers (73F, 115M, 42.7 ± 23.6 years) between the ages of 14 and 92 years. Of these, 81 volunteers from 20 to 85 years of age (28F, 53M, 47 ± 22.8 years) completed both the finger and leg dexterity protocols in order to evaluate dexterity systemically. Participants were excluded if they had pathology of the hand or a history of injury that prevented unrestricted use of their fingers or legs.

### Clinical populations

We used a sample of convenience from two clinical conditions known to affect hand function as a first exploration of the clinical utility of this paradigm. Our goal was not to diagnose or evaluate treatment, but simply collect cross-sectional data from patients suffering from these conditions. For these clinical groups, participants were excluded if they were undergoing treatment for injury or surgery and had not been released by their surgeon or physical/occupational therapist to participate in everyday ADL, had a concurrent injury or pathologic condition that caused pain or discomfort in the tested limb during physical activity and/or at rest, had clinical, surgical, physical, cognitive, or other conditions that may have prevented their ability to perform the tasks proposed in this study, including the clinical restriction decided by the surgeon or therapist, or were unable to complete the protocol.

The first clinical group, defined as patients treated for CMC OA, consisted of 33 female participants (65.81 ± 9.72 years, 42 hands) evaluated at an average of 40 months after treatment at Institut de la Main. The same surgeon (Caroline Leclercq) performed the treatments on all the patients. The CMC OA patients underwent one of four treatment types: ligament reconstruction with tendon interposition (LRTI) arthroplasty (21), trapeziectomy (TS) (22), non-surgical medical treatment (i.e., rehabilitation), and no treatment.

The second clinical group, defined as patients treated for PD, consisted of 16 volunteers (10M, 6F; 67.68 ± 8.5 years, 31 hands). All patients were treated at the USC Keck School of Medicine, Department of Neurology in the Parkinson’s Disease and other Movement Disorders Clinic.

### Strength–dexterity test

**Figure 1:**
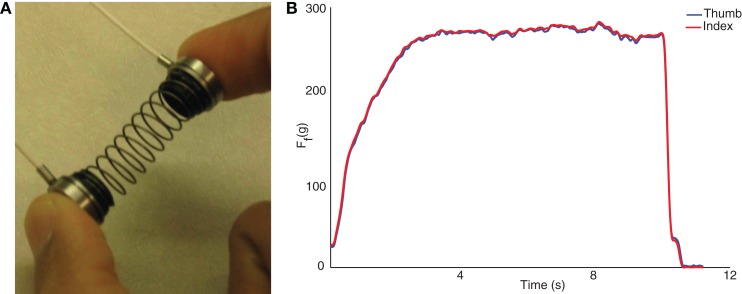
**The SD test (A) consists of compressing a compliant, slender spring prone to buckling, and sustaining the maximal level of compression for >5 s**. The pulps of the thumb and index finger press against miniature load cells. Sample data from spring compression are shown to the right **(B)**. The forces from the thumb and index finger, in gf, are averaged to calculate the maximal compression force.

The SD test is well described elsewhere (3–12). Briefly, it involves using the fingertips to compress as far as possible a slender spring, prone to buckling. This requires control of fingertip motions and force vectors at very low force levels (Figure 1A). It was conducted with a custom spring (Century Springs Corp., Los Angeles, CA, USA) outfitted with two miniature compression load cells (ELB4–10, Measurement Specialties, Hampton, VA, USA). The load cells were connected to a signal-conditioning box and USB-DAQ (National Instruments, Austin, TX, USA), sampled at 2000 Hz using custom Matlab (The Mathworks, Natick, MA, USA) software, and calibrated with a deadweight procedure. Participants were asked to compress the spring in a controlled way at their own pace to the point of maximal instability they can sustain (i.e., beyond which they felt it would slip out of their hand), and maintain that compression at a steady level for at least 5 s (Figure 1B) (9, 10). They were then to release in a controlled way at their own pace. After familiarization, at least 10 trials were performed for each test limb and the compression force was defined as the mean of the three maximal trials. Participants were allowed as many practice trials as needed to obtain steady state compression for the minimum required compression time of 5 s.

### Lower extremity dexterity test

**Figure 2:**
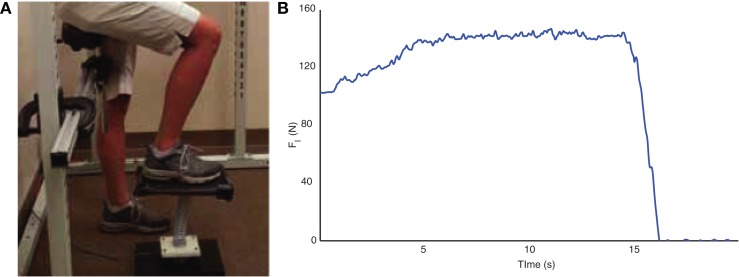
**The LED test (A) consists of pressing an appropriately scaled-up spring with the foot against the ground**. Compression forces, in N, are quantified with a load cell located under the spring. Sample data from spring compression are shown to the right **(B)**.

Similar to the SD test, the LED test is a single leg dynamic contact control task that is based on the ability of participants to compress a slender spring (19, 20, 23). The LED test device consists of a helical compression spring (Century Springs Corp., Los Angeles, CA, USA) mounted on a single-axis force sensor (Transducer Techniques, Temecula, CA, USA) affixed to a stable base with a 15 cm × 30 cm platform affixed to the free end (Figure 2A). Participants were positioned in an upright partially seated posture on a bicycle saddle intended to stabilize the body and minimize the extraneous use of the contralateral limb and upper extremities during testing. A computer monitor provided visual force feedback of the vertical force (19, 20, 23). As with the SD test, participants were instructed to slowly compress the spring with their foot with the goal to raise the force feedback line as high as possible and maintain that compression for at least 10 s (Figure 2B). After familiarization, between 10 and 20 trials were performed for each test limb (19, 20, 23) and the compression force was defined as the mean of the three maximal trials. Participants were allowed as many practice trials as needed to obtain steady state compression for the minimum required compression time of 10 s.

### Data analysis and variable descriptions

The dependent variables for the SD and LED tests are defined in Table 1. Linear regressions, two-tailed *t*-tests, and analysis of variance (ANOVA) were applied to the data set, as appropriate, to identify and quantify the relationships between test performance, age, gender, and dominance and to compare performance between clinical and control populations. Significance was set at *p* < 0.05 for all analyses. Matlab R2013a and SPSS version 22 (IBM, Armonk, NY, USA) were used for these analyses.

**Table 1:** **Definition of variables used in analyses**.

Variable	Symbol	Description
Finger compression force	*F_f_*	Mean compression force during the hold phase of the SD test (units: gf)
Finger force velocity	${\dot{F}}_{f}$	Mean of the absolute value of the first time derivate of compression force during the hold phase of the SD test (units: gf/s)
Finger force acceleration	${\ddot{F}}_{f}$	Mean of the absolute value of the second time derivate of compression force during the hold phase of the SD test (units: gf/s^2^)
Finger force RMS	RMS*_f_*	Magnitude of the mean of the force dispersions during the hold phase of the SD test (units: gf)
Leg compression force	*F_l_*	Mean compression force during the hold phase of the LED test (units: N)
Leg force velocity	${\dot{F}}_{l}$	Mean of the absolute value of the first time derivate of compression force during the hold phase of the SD test (units: N/s)
Leg force acceleration	${\ddot{F}}_{l}$	Mean of the absolute value of the second time derivate of compression force during the hold phase of the SD test (units: N/s^2^)
Leg force root-mean square (RMS)	RMS*_f_*	Magnitude of the mean force dispersions during the hold phase of the SD test (units: N)

*Note that force magnitudes for the finger and leg tasks (cf. Figures 1 and 2) are two orders of magnitude apart. Therefore, we use the SI units of gf and N, respectively, to accommodate those differences*.

## Results

### Overview

The ANOVA results are summarized in Table 2 and discussed in detail in this section. We report strong age and gender effects in leg and finger compression force in healthy participants. Furthermore, we report strong effects of clinical condition (both CMC OA and PD) on the force velocity, acceleration, and RMS of the SD test. Interestingly, we report no differences in any variable between the dominant and non-dominant sides of control participants, patients diagnosed with CMC OA, and between self-reported affected and unaffected sides of patients diagnosed with PD.

**Table 2:** **Summary of multifactor ANOVA results**.

Variable	Age	Gender	Side	Clinical condition
Finger compression force (*F_f_*)	*p = 0.017^a^	*p = 0.021^a^	Control: *p* = 0.461^a^	*p* = 0.081
			PD: *p* = 0.784	
			CMC OA: *p* = 0.327	
Finger force velocity $\left({\overset{˙}{F}}_{f}\right)$	*p = 0.048^a^	*p* = 0.542^a^	Control: *p* = 0.408^a^	*p < 0.001
			PD: *p* = 0.668	
			CMC OA: *p* = 0.786	
Finger force acceleration $\left({\overset{¨}{F}}_{f}\right)$	*p* = 0.061^a^	*p* = 0.158^a^	Control: *p* = 0.672^a^	*p < 0.001
			PD: *p* = 0.725	
			CMC OA: *p* = 0.849	
Finger force RMS (RMS*_f_*)	*p* = 0.880^a^	*p* = 0.989^a^	Control: *p* = 0.183^a^	*p < 0.001
			PD: *p* = 0.696	
			CMC OA: *p* = 0.755	
Leg compression force (*F_l_*)	*p = 0.005	*p = 0.002	*p* = 0.295	–
Leg force velocity $\left({\overset{˙}{F}}_{l}\right)$	*p* = 0.595	*p* = 0.536	*p* = 0.945	–
Leg force acceleration $\left({\overset{¨}{F}}_{l}\right)$	*p* = 0.519	*p* = 0.441	*p* = 0.872	–
Leg force RMS (RMS*_l_*)	*p* = 0.532	*p* = 0.135	*p* = 0.237	–

*^a^Indicates transformed data set*.

*indicates significance level of 0.05.

The results from the linear regression analyses of compression force with respect to age are summarized in Table 3. We report significant increases in compression force in both the finger and leg in healthy participants under the age of 40, and vice versa for those over the age of 40 years – but as clarified in the Section “Discussion,” this effect is not always seen when separating subjects by gender. Furthermore, there were greater decreases in force with age in the clinical groups compared to unimpaired participants.

**Table 3:** **Summary of linear regressions of compression force with age results**.

Variable	Controls <40 years			Controls >40 years			Clinical participants	
	Males	Females	All	Males	Females	All	CMC OA	PD
Finger compression force (*F_f_*)	*p* = 0.328	*p* = 0.316	*p = 0.019	*p* = 0.09	*p = 0.008	*p = 0.002	*p < 0.001	*p < 0.001
Leg compression force (*F_l_*)	*p = 0.001	*p* = 0.09	*p < 0.001	*p* = 0.055	*p* = 0.076	*p = 0.007	–	–

### Finger SD test with control subjects in the self-reported dominant hand

We tested for the effects of age and gender on finger dexterity in the self-reported dominant hand of 147 healthy individuals between the ages of 20 and 88 years. When needed, some variables (*F_f_*, ${\dot{F}}_{f}$, ${\ddot{F}}_{f}$, and RMS*_f_*) were transformed using the natural logarithm function to meet the assumptions of normality required for parametric statistics. As shown in Table 2, an ANOVA with finger compression force as the dependent variable and age and gender as factors performed on the transformed data revealed a significant effect by both age (*p* = 0.017) and gender (*p* = 0.021). Furthermore, we report no gender effects on the compression dynamics (${\dot{F}}_{f}$, ${\ddot{F}}_{f}$, and RMS*_f_*) and no age effects on force accelerations and RMS, but age does affect the finger force velocity (*p* = 0.048) (Table 2).

**Figure 3:**
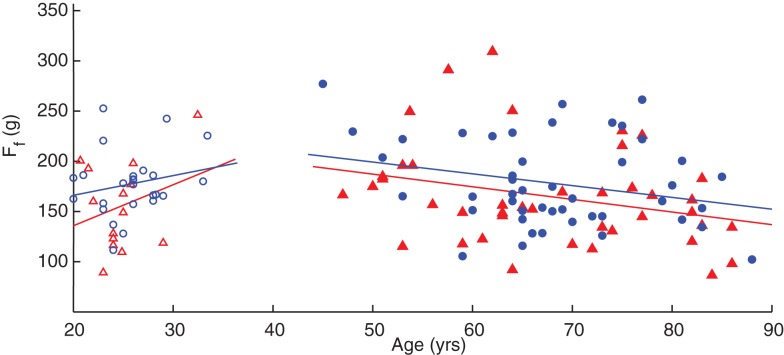
**Linear regression of finger compression force with respect to age**. Younger adults (empty symbols) tended to show an increase in compression force while older adults (filled symbols) showed a decrease. Male participants (blue circles) tended to have greater values than females (red triangles) as indicated by the position of the fit lines. SeeTable 3.

A linear regression of finger compression force with respect to age, grouped by gender, is shown in Figure 3. Without accounting for gender, adults under the age of 40 years have an increase in finger compression force with age (*p* = 0.019) while adults over 40 have a decrease in force with age (*p* = 0.002). When the groups are separated by gender, however, the increases in compression force in younger males and females and decreases in older males are no longer significant (Table 3). Note the offset in regression lines, which agrees with the significant on the gender effect on compression force as per the ANOVA.

### Finger SD test with clinical subjects

**Figure 4:**
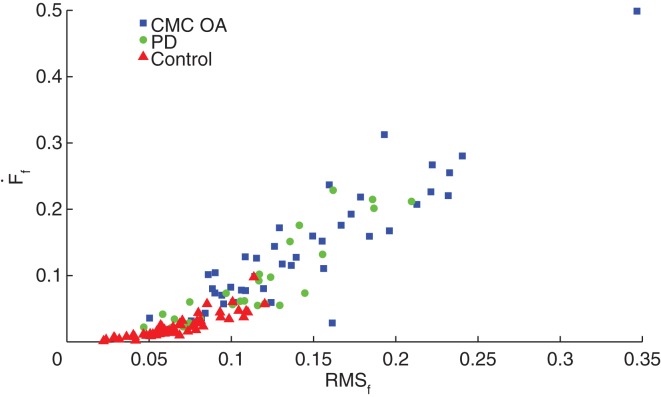
**Dynamic characteristics of the SD test**. Control participants (red triangles) had significantly greater stability during SD compression compared to patients with CMC OA (blue squares) and PD (green circles).

We compared performance on the SD test (*F_f_*, ${\dot{F}}_{f}$, ${\ddot{F}}_{f}$, and RMS*_f_*) between clinical patients diagnosed with either CMC OA or PD and a subset from our dataset of 29 healthy, age-matched volunteers (10M, 19F; 65.6 ± 9.7 years, 48 hands) with no history of hand injury or disease or neurological disorder. Interestingly, we found no significant differences in finger compression force among groups, however we found differences between the clinical and control groups in compression dynamics (${\dot{F}}_{f},{\ddot{F}}_{f}$, and RMS*_f_*) during the sustained compression as illustrated in Figure 4. We found no differences in compression dynamics between the PD and CMC OA groups; however, both groups showed significant differences from the control participants (*p* < 0.001), indicating distinctly different dynamical behavior during manipulation in these clinical populations (Table 2).

**Figure 5:**
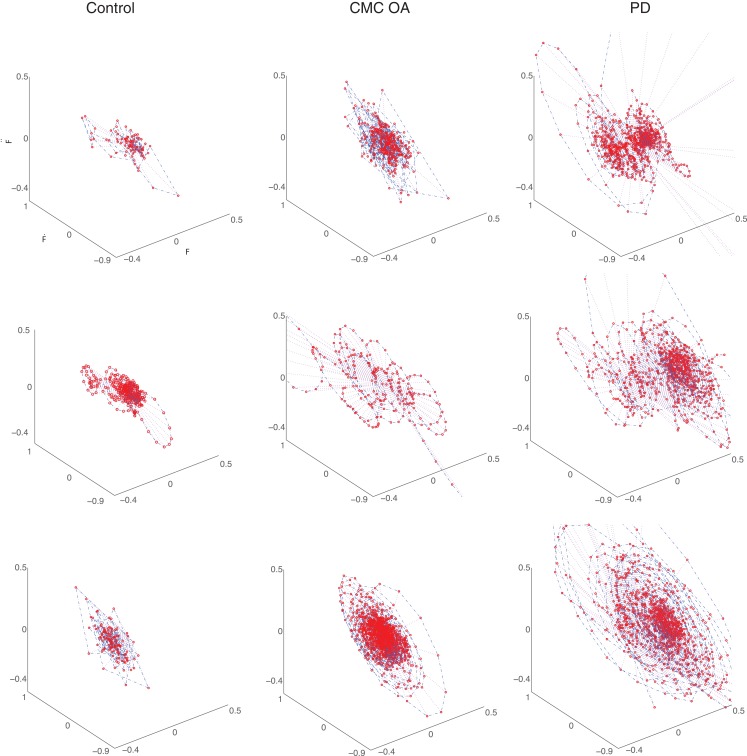
**Representative phase portraits of three participants from each group (ages between 70 and 75 years): healthy control subjects (first column), participants diagnosed with CMC OA (second column), and participants diagnosed with PD (third column)**. The clinical subjects exhibit greater dispersion in the phase portrait than the control subjects.

Additionally, as in Ref. (9, 10, 13), we characterized the force dynamics during the sustained compression by plotting the phase portraits of *F_f_* versus ${\dot{F}}_{f}$ versus ${\ddot{F}}_{f}$ (Figure 5). The character of the phase portrait was quantified by the mean Euclidean distance from the origin per unit time (9, 10, 13). A greater Euclidean distance is suggestive of weaker corrective actions by the neuromuscular controller enforcing the sustained compression (9, 10, 13). There are clear differences in the phase portraits of the control and clinical participants, with greater dispersion associated with the clinical groups.

**Figure 6:**
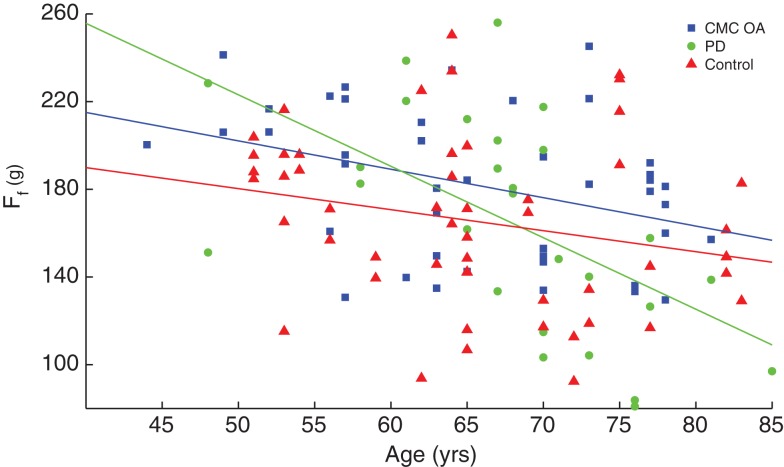
**Comparison of rate of decline between clinical and control populations**. Finger compression force was plotted against age and revealed that the clinical groups (PD and CMC OA, green circles and blue squares, respectively) had a greater rate of decline with age than control participants (red triangles).

We also performed linear regressions of finger compression force versus age in these three populations, which revealed that individuals with CMC OA and PD showed greater rates of decline compared to control subjects (*p* < 0.001), Figure 6. Patients with CMC OA and PD had average rates of decline of −1.4 and −2.7 gf/year, respectively, compared to −0.86 gf/year in control participants (Table 3).

To further expand the analysis and investigate the effect of laterality, we compared performance on the self-reported affected hand to the unaffected hand in a subset (*n* = 8) of the PD group. An ANOVA revealed no effect of side in any variables (*F_f_*, ${\dot{F}}_{f},{\ddot{F}}_{f}$, and RMS*_f_*; Table 2). We performed a similar analysis on the self-reported dominant and non-dominant hands of a subset of the CMC OA group (*n* = 17) and report no effect of laterality in any variable (*F_f_*, ${\dot{F}}_{f},{\ddot{F}}_{f}$, and RMS*_f_*; Table 2).

### Leg LED test with control subjects in the right leg

Mirroring the work on finger dexterity, we also tested for effects of age, gender, and dominance on leg dexterity in the right leg of 188 healthy individuals from 14 to 92 years. In order to account for the age and gender effects on body weight, which may influence leg compression force, we included body mass index (BMI) in the analysis. The data were normally distributed, and an ANOVA with leg compression force as dependent variable, age and gender as factors, and BMI as a covariate showed that compression force is strongly affected by both age (*p* = 0.005) and gender (*p* = 0.002; Table 2), but not by BMI (*p* = 0.198). Furthermore, ANOVA on the force dynamics (${\dot{F}}_{l},{\ddot{F}}_{l}$, and RMS*_l_*) during sustained compression showed no effect of gender, age, or BMI.

**Figure 7:**
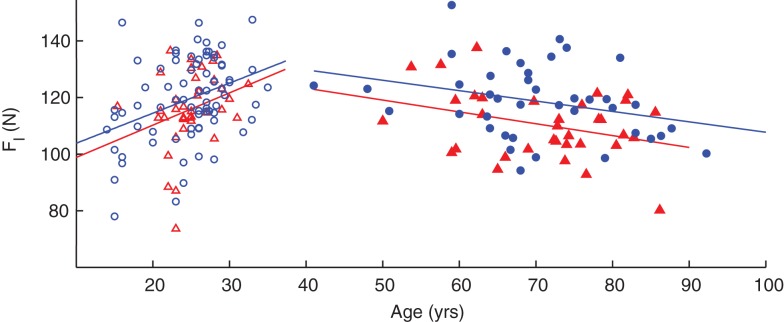
**Age- and gender-related changes in leg compression force**. Regressions against age indicated an increase in younger adults (empty symbols) and a decrease in older adults (filled symbols). Male participants (blue circles) tended to have greater values than females (red triangles) as indicated by the position of the fit lines.

Linear regressions of leg compression force versus age revealed significant increases in force in adults under the age of 40 (*p* < 0.001) and decreases in participants over 40 years (*p* = 0.007). However, when separated by gender, increase in compression force in young females and decreases in older males and females are no longer significant (Table 3). As with the hand, there are increases in compression force with respect to age in younger adults and decreases in older adults; and the regression lines of male participants are slightly shifted above those of females, corroborating the ANOVA results that compression forces for male participants tended to be greater on average than that of female participants when using age as a factor (Figure 7). Note that in these subjects we only tested one leg, the right leg, for expediency because the effect of leg dominance was explored in a different subset of subjects (see below).

### Dexterity across both fingers and legs

**Figure 8:**
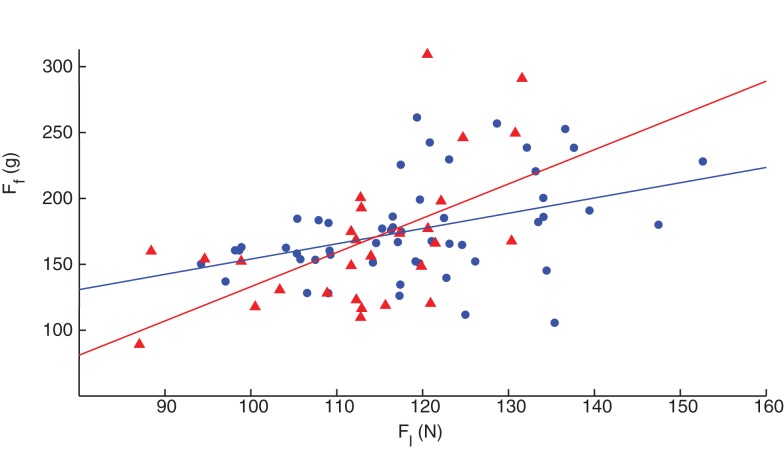
**Correlation of finger and leg dexterity**. Both male (blue circles) and female (red triangles) participants showed significant association between finger and leg compression force in the self-reported dominant limb, with females exhibiting higher correlation than males, ρ = 0.529 and 0.403, respectively.

Finally, we explored dexterity across the upper and lower extremities by comparing SD and LED performance in both fingers and legs of 81 healthy volunteers between the ages of 20 and 85, each labeled as self-reported dominant or non-dominant (Figure 8). Surprisingly, ANOVA (in this case a repeated measures ANOVA given that we collected finger and leg data in the same subjects) revealed no effects of laterality (i.e., dominant versus non-dominant) for any variable, when controlling for gender and age in these participants (Table 2). However, we found statistically significant (*p* < 0.001) Pearson’s product–moment correlation of ρ = 0.458 between finger and leg compression forces in all subjects. When separating them by gender, the Pearson’s product–moment correlation was higher in females (ρ = 0.529, *p* = 0.004, *n* = 28) than in males (ρ = 0.403, *p* = 0.003, *n* = 53).

## Discussion

There are multiple definitions for, and connotations of, the concept of dexterity. In a series of recent publications using the SD paradigm, we have argued that quantifying the sensorimotor ability to stabilize objects with the fingertips is a valid definition of one aspect of finger dexterity (3–10). By focusing on how the fingertips act on an object by dynamically regulating the magnitude and direction of fingertip forces, we can quantify important features of using precision pinch (or tip-to-tip, or pincer grasp) to manipulate objects. Therefore, the purpose of this comparative cross-sectional study was to quantify how these features of dexterous manipulation are affected by age, gender, and disease. We have previously attributed the sensitivity of the SD test to detect functional changes among both healthy and clinical populations across the life span to its ability to focus on the sensorimotor function of the isolated CNS-limb system without the confounds of visual acuity, whole-arm function, or finger strength (3, 5, 6, 9–12). Furthermore, it has allowed the detection and identification of specific and context-sensitive brain circuits for dynamic control of the fingers (4, 7, 8). Those prior findings inform our interpretation of our important results now quantifying the effects of gender, age, and disease.

### Effect of age

Our results corroborate the effect of age we have reported for finger dexterity in young children and adolescents (10), and older adults (13). However, we extend those results in crucial ways. It is important to note that our prior work (9) revealed no significant changes in dexterous manipulation in middle age and therefore, we used samples of convenience (college-aged students and older control subjects for comparison to clinical populations of interest), which resulted in an under sampling of subjects between 35 and 50 years of age, but does not affect the results we report. First, we emphasize our study of adults starting at 20 years of age, where we continue to see an improvement in young adulthood. In an earlier study, we report the strong association between improvements in finger compression force and compression dynamics with maturation of the brain in children and adolescents (10). To our knowledge, this is the first report of continual improvement of dexterity into young adulthood after the age of 20. The continual behavioral improvements we see here are, therefore, credibly associated – at least in part – with such neural maturation and have important clinical implications for rehabilitation. For example, traumatic injuries [such as spinal cord injury in males (24) and anterior cruciate ligament (ACL) tears in females (17)] are most prevalent in young adults. Our results indicating the presence of motor learning and neural plasticity in early adulthood suggest that these individuals would naturally have a propensity to respond to therapy better than older adults. Similarly, our results now come from 147 adults from 20 to 88 years of age. These include 108 subjects not previously analyzed and 39 from our previous reported pool of 98 subjects (13). This was critical to reveal the gender effect in finger compression not previously significant (see below and Table 2), and now confirm what was a near significant effect of age on finger force dynamics hinted at in our previous work (6, 9, 10, 13), Table 2.

While we also corroborate the finding that finger dexterity begins to decline in middle age (13), this study goes on to reveal differences in that decline in individuals aging with a disability. We find that one condition (PD) exhibited a rate of decline two times greater than another (CMA OA), and three times greater than non-symptomatic control subjects (Figure 7). This has important implications to the differential role in which different disease mechanism produce disability (see below). Aside from the clinical details we discuss below, the idea that finger dexterity is an indicator of the integrity of the sensorimotor system (3), together with the idea that loss of dexterity in older adults is not linked to muscular weakness (13) or BMI, leads to the implication that in older adults the ability of the nervous system to respond to therapy is increasingly muted.

In our prior work (10) we have noted that, in parallel with the development of the ascending and descending pathways between brain and hand, there are striking developmental processes taking place in the brain gray and white matter during childhood up to adolescence, e.g., expansion of the white matter and pruning of the cortical gray matter (25–30). Ehrsson et al. (31) demonstrated that there is greater activity in the fronto-parietal sensorimotor areas during the control of smaller forces than larger forces, with control of larger forces associated with increased activity in the M1 region. Fronto-parietal regions demonstrate significant developmental changes in the adolescent years (28, 29, 32), and the pruning of the gray matter occurs later in the frontal and parietal areas (33) than in M1. These associations between the development of cortical neural networks, including ascending and descending pathways on one hand, and the dexterity measured by our method are, of course, mostly empirical and speculative. Our results now raise the possibility that these processes continue into young adulthood. Moreover, they also seem to be reversed (or counteracted) by the mechanisms of aging in a way that is behaviorally measurable, in a way that has important clinical and therapeutic implications.

### Effect of gender

The effect of gender on motor skill is not well documented, necessarily predictable, or expected in dynamic finger function – contrary to the well known effect of gender on muscle strength or BMI. Given those differences in strength across genders, we designed our test of dynamic sensorimotor function to require only very low levels of force (<300 gf). We have reported hints of a gender effect on dexterity in typically developing children (6) – which may have been colored by a test protocol that tended to require large forces. However, these new results now establish without a doubt that females exhibit lower ability to control instabilities with the fingertips than males at any age. The literature does not report consistent gender effects, and the issue remains very much debatable (6, 15, 18, 34). Our results add to this literature by providing a new example of performance differences between women and men.

Given that we have found the SD paradigm to be informative of local and systemic neuromuscular mechanisms [e.g., brain maturation, muscle contractile speeds, functional brain connectivity and networks, etc. (3–10)], this clear gender effect is remarkable as it strongly suggests those sensorimotor differences in women are a function of specific mechanisms at the level of the muscles, spinal cord, and/or brain. This leads directly to testable hypotheses at each of these hierarchical levels. For example, does the excitability of motoneuron pools during the control of unstable forces change differently in men versus women? What are the roles of hormonal cycles in the general excitability and controllability of the sensorimotor system? Are there differences in brain connectivity in sensorimotor areas across genders as is now reported for cognitive areas? There is a growing consensus that male brains are structured to facilitate connectivity between perception and coordinated action, whereas female brains are designed to facilitate communication between analytical and intuitive processing modes (35). Our methodology now allows us to systematically interrogate those differences in the context of the functionally critical areas of dexterity.

### Effect of clinical condition

Our study also raises the similarly noteworthy question of why a condition that is presumably purely orthopedic (i.e., CMC OA) produces deficits in dynamic manipulation – and accelerated losses with age – comparable to those in a purely neurological condition (i.e., PD). Both the CMC OA and PD groups displayed significant differences (*p* < 0.001) in the compression dynamics (${\dot{F}}_{f},{\ddot{F}}_{f}$, and RMS*_f_*) compared to the control participants (Figure 4), although no differences in compression force. That is, all three populations were able to compress to the same amount, but not in the same way. Similarly, detailed visualization of the finger force dynamics during compression via phase portraits (Figure 5) shows subjects with CMC OA and PD tend to demonstrate weaker correction strategies. The greater amount of dispersion in the phase portraits of clinical patients suggests a compromised ability to execute corrections, or a different neural control strategy toward instability, not seen in control subjects (10, 13). Whether these differences in neural control, or the mechanisms of executing neural control, are similar or different in CMC OA and PD remains an open question.

These results also challenge the notion that CMC OA is a strictly orthopedic condition given that we now see it produces sensorimotor deficits. The link between a disease of articular cartilage and deficits in sensorimotor integration capabilities is underappreciated and understudied in the literature. To elaborate, Figure 4 illustrates that the CMC OA and PD populations are essentially indistinguishable when plotting finger force velocity versus finger force RMS. These results raise the question, what is it about chronic pain and damage to the joint that leads to changes in sensorimotor capabilities? Others have begun to speak about this and a picture is now emerging showing that chronic pain leads to reorganization of brain circuits. For example, subacute low back pain induces changes in connectivity and functional reorganization of the insula and sensorimotor cortex, even after only 1 year with moderate pain (36). Also, spontaneous pain due to knee OA is known to engage brain regions distinct from those activated by pressure-evoked pain, specifically prefrontal-limbic structures (37). The presence of acute pain will naturally compromise function – but we now see that chronic pain also affects the performance of a dexterous task even if it requires very low forces and does not elicit pain. Our prior work suggests these deficits are credibly attributable to structural or functional changes in portions of the nervous system responsible for the neural control of dexterity.

At the other end of the clinical spectrum, PD starts out as a purely neurological degenerative disease characterized by upper and lower extremity rigidity, tremor, bradykinesia, and/or postural instabilities (38, 39). Our prior work has shown that the cortical networks associated with controlling instabilities in dexterity can involve the basal ganglia (8), where degeneration of dopamine-producing cells plays a central role in PD (39). Thus it is expected that we would detect deficits in sensorimotor function and, in turn, dexterous manipulation in this population. However, our results allow us to go deeper than this. They allow us to, for the first time, (i) systematically quantify behavioral deficits in PD and other neurological conditions, (ii) disambiguate the contributions of different elements of the neuromuscular system to these deficits, and (iii) easily and objectively quantify the effectiveness of different treatment regimens (e.g., absorption of medication or titration of deep brain stimulation level) during the daily – and even hourly – fluctuations in motor deficits in PD that traditional measures cannot. However, it is also critical to note that PD leads to significantly greater rates of decline of dexterity with age when compared to healthy aging or with patients diagnosed with CMC OA. This highlights the neurodegenerative nature of the disease, and underscores the need to quantify the effects of PD on sensorimotor processing and dexterous manipulation to better understand its neurodegeneration and treatment.

How do our results speak to ADLs? The SD paradigm falls clearly within the Body Functions and Structure Components of the International Classification of Function [ICF (40)]. Understanding the link between SD performance and the Activity Limitations and Participation Restriction Components of the ICF requires further research. But as of now, we can say that the SD paradigm is likely very informative of systemic mechanisms that make dexterous function possible – as argued throughout the Section “Discussion.” That is, the SD paradigm reflects the potential to execute ADLs without the confounds of functional adaptations that mask the detrimental effects of disease. A clear example for the upper extremity is that of manipulating small and/or deformable objects such as beads or squeezing lemons, respectively. In both these cases, the manipulation task is unstable in the same sense that the SD paradigm specifies: they require accurate dynamical regulation of the magnitude and direction of fingertip forces and motions (9, 10, 13). For the lower extremity, we have proposed that the SD paradigm may explain the risk of injury or falls (19, 20, 23) because the regulation of dynamical interactions with the ground is critical to locomotion and many sports activities, as mentioned above.

### Systemic versus limb-specific dexterity

Another fundamental aspect of this work is that we extended the concept of finger dexterity to limbs in general. We use the same definition of dexterity to quantify the sensorimotor ability of the leg to regulate dynamical interactions with the ground in a subset of our participants. In the context of lower extremity function, the LED test evaluates the ability of the sensorimotor system to control an unstable ground contact with the isolated leg; and avoids potential confounds often found in gait, posture, and balance studies such as vestibular function, visuo-spatial perception, strength, whole-body balance, locomotor confidence, and inter-limb coordination. Clearly, our aim is not to study locomotion, but to focus on the fundamental sensorimotor capabilities of the leg. Further work is needed to establish its relationship to whole-body gait, posture, and balance capabilities. Nevertheless, our recent work on the lower extremity has demonstrated the validity and reproducibility of the LED test as a metric of dynamic leg function, and its correlation to whole-body agility. It has also clearly detected differences between young men and women (19, 20, 23). As in the case of the fingers (6), we have shown that the LED test quantifies a previously unrecognized functional domain related to dexterity of the isolated leg that cannot be seen as simply a covariate of available functional tests of strength, gait, or balance (41). Here we extend that prior work on leg dexterity by measuring the same set of variables as for the finger in 188 healthy volunteer participants (Tables 1–3). To our knowledge, this is the first comparison of finger versus leg dexterity that allows us to distinguish between systemic and limb-specific sensorimotor capabilities. Interestingly, we find similar effects of age and gender in both finger and leg dexterity.

The age and gender effects on leg compression force (Figure 7; Table 3) naturally suggest that the same neural mechanisms and networks for the fingers (discussed above) are at work in the leg to some extent. Traditionally we have come to think of “dexterity” as specific to fingers [e.g., Ref. (42– 45)], and surely some features are. Phylogenetically speaking, however, legs evolved earlier and for the same purpose: to produce dynamical interactions with the ground. Thus, the prior existence of neural circuits to regulate instabilities in ground contact during quadruped gait and brachiation likely served as the foundation from which specializations evolved for manipulation in the human hand. Therefore, our discussions above about the neurophysiological bases of age and gender effects apply here as well. However, there are also important differences. We found no age and gender effects on compression dynamics (${\dot{F}}_{l},{\ddot{F}}_{l}$, and RMS*_l_*), and most of these effects are far from significant even in this relatively large sample size (Table 2).

These similarities and differences between finger and leg dexterity, as quantified by the SD and LED tests, suggest the existence of specialized mechanisms for systemic versus limb-specific dexterity. First, it is clear that these results compel us to study in detail the neurophysiological bases of leg dexterity in health and disease, to at least to the level we have for the fingers. Moreover, the multiple time scales and latencies with which these dynamical tasks need to be controlled suggest a hierarchical organization of neural control, in agreement with current thinking (46–48). However, we must not be content with this generalization. Future work must leverage available techniques [e.g., electromyography (EMG), fMRI (7, 8), Hoffmann-reflex, transcranial magnetic stimulation (TMS), coherence analysis (49), EMG-weighted averaging (50)] in specific and well-directed studies to disambiguate among peripheral, spinal, and cortical contributions and mechanisms of dexterity. The SD paradigm allows such studies for the legs as it has for the fingers.

Second, our findings about leg dexterity nevertheless have immediate utility, both scientifically and clinically. Understanding the orthopedic and neurological effects of aging with a disability on quality of life is now emerging as an important public health issue (51–55) of immediate interest is the study of leg dexterity in patients with PD, where shuffle gait, ataxia, and bradykinesia are common – and the SD paradigm combined with clinical outcome measures and the techniques mentioned above will serve to clarify the mechanisms enabling leg dexterity and their neuroanatomical and functional hierarchy. Similarly, it is important to follow up with studies in patients with hip or knee OA, where we can begin to understand the effects of chronic pain on locomotor abilities both because OA is so prevalent, and because gait deficits that lead to falls in the elderly are a pressing public health problem (56).

In addition to providing insight into the nature of sensorimotor dysfunction in clinical populations, the fact that the LED test is able to discern gender differences (Figure 7; Table 2) may provide insight into why young women have a much greater likelihood of non-contact ACL tears than men (57). Though the reasons are not clear, some theories include differences in knee alignments, ligament laxity, hormone levels, muscle strength and conditioning, and neuromuscular control (17, 20). The clearly reduced dexterity we report in young women (both in fingers and legs) expands on previous results (20) with a smaller sample size where gender differences in dexterity were used to provide a neuromuscular explanation for the higher incidence of ACL tears and reduced agility in young female athletes. Moreover, given that we now show that these gender differences in leg dexterity are present throughout the lifespan also speaks to the fact that women over the age of 65 have a disproportionately greater occurrence of unintentional falls than men (16, 58). Future work will include identifying those with reduced leg dexterity who may have a greater risk for ACL tears or falls and would benefit from preventative neuromuscular training programs.

Interestingly, we saw no clear effect of limb dominance on finger and leg dexterity in the subset of 81 participants who completed the SD paradigm with all four limbs. After all, voluntary fine-motor tasks such as writing, cutting, catching, and kicking exhibit strong effects of laterality. In fact, there is a multitude of evidence supporting both functional (e.g., strength and motor control) and anatomical differences at the cortical level between dominant and non-dominant limbs (15, 59–64). It is reported that long-term preferential use of muscles results in a higher percentage of type 1 muscle fibers in the dominant hand and, in turn, changes in motor unit firing behavior (61). Furthermore, imaging studies have shown that the hemisphere contralateral to the dominant hand demonstrates more efficient motor control at lower activation levels and less crosstalk than the non-dominant hemisphere (62, 63). One potential explanation is that we simply did not have enough subjects to demonstrate that latent effect, much as we did not find an age or gender effect in this same group of 81 subjects spanning multiple ages. This mirrors our prior work where we were not able to detect gender effects for the upper extremity in studies with smaller sample sizes (9). What is more striking, however, is that larger numbers may be needed to detect an effect of limb dominance, if it is even present.

Our lack of detection of limb dominance nevertheless raises important questions. As mentioned recently, it is likely that hemispheric specialization emerged to accommodate increasing motor complexity of tasks during primate evolution. That is, instead of the non-dominant limb being a lesser analog of the dominant limb, Sainburg and colleagues (65) have proposed an alternative view that motor lateralization reflects proficiency of each arm for complementary functions in response to distinct movement control mechanisms associated with specific unimanual tasks. We speculate that the lack of effect of dominance suggests that the SD and LED tests reveal and quantify subcortical mechanisms for dynamical function that are not influenced by hemispheric differences – in accordance with theories of hierarchical neural control and phylogenetic development of the nervous system. There is evidence of subcortical contributions to motor control (i.e., dexterity) independent of limb dominance. In this hierarchical view of motor control, the cerebellum, basal ganglia, spinal cord, etc. are essential to executing and regulating motor function. In agreement with Sainburg and colleagues (65), we speculate that hand (or leg) dominance is therefore likely a late arrival to the motor repertoire in humans that affects fine-motor tasks but not “low-level” stabilization mechanisms tested by the SD paradigm. This is supported by recent studies using Blood Oxygenation Level-Dependent (BOLD fMRI) to evaluate how hand dominance and task difficulty affect activation levels at the spinal cord (66) level. They found significant differences in spinal cord activation levels when performing simple unilateral tapping tasks with the dominant and non-dominant hands – but they found no effect of hand dominance during a more complex unilateral tapping task. The SD paradigm may be engaging these systemic hierarchically common circuits to all limbs independently of cerebral lateralization. A clinical consequence of this may be the fact that we did not see differences across the self-reported affected versus unaffected hand in patients with PD – although this requires further clinical investigations with greater numbers of individuals.

How does this concept that dexterity requires both subcortical and cortical mechanisms agree with or revise current thinking? Very briefly, the literature on cortical involvement in dexterous manipulation is large [e.g., the reviews in Ref. (45, 67, 68)]. Our own fMRI studies agree with many others suggesting direct cortical involvement by showing the SD paradigm can systematically interrogate brain function for dexterous manipulation, which exhibits differential activity across cortical networks depending on the level of difficulty and behavioral goals of the task (4, 7, 8). We have also proposed the likely evolutionary advantage of the monosynaptic corticospinal tract to manipulation by enabling the time-sensitive transitions from the control of motion to the control of static force (5); and that the competition between descending commands for manipulation likely involves the phylogenetically older reticulospinal and the newer corticospinal tracts (69). However, our results here compel us to confront several inconvenient facts to the cortico-centric view of the neural control of the hand including time delays, our evolutionary history, and clinical symptomatology. These issues can be resolved by paying more attention – and due credit – to subcortical mechanisms. For example, many dynamic manipulation tasks (such as stabilization in the SD paradigm) occur at time scales for which spinal–cortical–spinal delays would compromise closed-loop control. Neural control must, therefore, involve motoneuronal modulation by the spine in human and non-human primates to some extent (70, 71). In fact, neuroanatomists and electrophysiologists since the time of Sherrington have sought to map the circuitry in the spinal cord (72) to understand the spinally mediated excitation–inhibition mechanisms that enable voluntary function [e.g., Ref. (73, 74)] – and produce the clinical symptomatology of, for example, spastic hypertonia present in many neurological disorders including stroke, traumatic brain injury, cerebral palsy, multiple sclerosis, and spinal cord injury [e.g., Ref. (75) and references therein]. Therefore, much as Lemon has written “it may be too sweeping a generalization to suggest that cortico-motoneuronal connections are the *sine qua non* of independent digit movements” (70), our results indicate that it may be too sweeping a generalization to suggest that cortical mechanisms are the *sine qua non* of dexterity. Once again, this compels future work to disambiguate among peripheral, spinal, and cortical contributions and mechanisms of finger and leg dexterity.

Finally, this is the first time that to our knowledge a same paradigm is used to quantify *both* finger and leg dexterity. We report their correlation in Figure 8, indicating that the sensorimotor system may have a combination of systemic versus limb-specific mechanisms, although the contribution of each remains unclear. The fact that this correlation is greater in female than in male participants (ρ = 0.529 versus ρ = 0.403, respectively) suggests a much greater systemic component in women. We speculate that dexterity is actually the sum of two components: the basic systemic, plus the limb-specific. The stronger systemic component in women may then suggest that men are able to add more of the limb-specific component and thus show less correlation overall. What could be the causes of this added plasticity for limb-specific dexterity in men? In addition to genetically imposed dimorphism (e.g., nature), sociobiological elements (e.g., nurture) such as differential exposure to physical activity, cultural biases, social expectations, etc., may play a role in the development and learning of motor function (76). Thus, the differences in dexterity across genders that we report, and in brain connectivity that others report, may be – at least in part – its phenotypical neurobiological consequence.

## Conflict of Interest Statement

Francisco J. Valero-Cuevas holds US Patent No. 6,537,075 on some of the technology used, but has no active or pending licensing agreements with any commercial entity. All other authors report no conflicts of interest.
